# Boosting Polarization Switching-Induced Current Injection by Mechanical Force in Ferroelectric Thin Films

**DOI:** 10.1021/acsami.1c04912

**Published:** 2021-05-26

**Authors:** Fengyuan Zhang, Hua Fan, Bing Han, Yudong Zhu, Xiong Deng, David Edwards, Amit Kumar, Deyang Chen, Xingsen Gao, Zhen Fan, Brian J. Rodriguez

**Affiliations:** † School of Physics, University College Dublin, Belfield, Dublin D04 V1W8, Ireland; ‡ Conway Institute of Biomolecular and Biomedical Research, University College Dublin, Belfield, Dublin D04 V1W8, Ireland; § Guangdong Provincial Key Laboratory of Functional Oxide Materials and Devices, Southern University of Science and Technology, Shenzhen 518055, People’s Republic of China; ∥ Institute for Quantum Science and Engineering, and Department of Physics, Southern University of Science and Technology, Shenzhen 518055, People’s Republic of China; ⊥ Department of Materials Science and Engineering, Southern University of Science and Technology, Shenzhen 518055, People’s Republic of China; # Institute for Advanced Materials and Guangdong Provincial Key Laboratory of Optical Information Materials and Technology, South China Academy of Advanced Optoelectronics, South China Normal University, Guangzhou 510006, People’s Republic of China; ∇ Centre for Nanostructured Media, School of Mathematics and Physics, Queen’s University Belfast, Belfast BT7 1NN, U.K.

**Keywords:** ferroelectric, BiFeO_3_, injected current, mechanical force, FeRAM

## Abstract

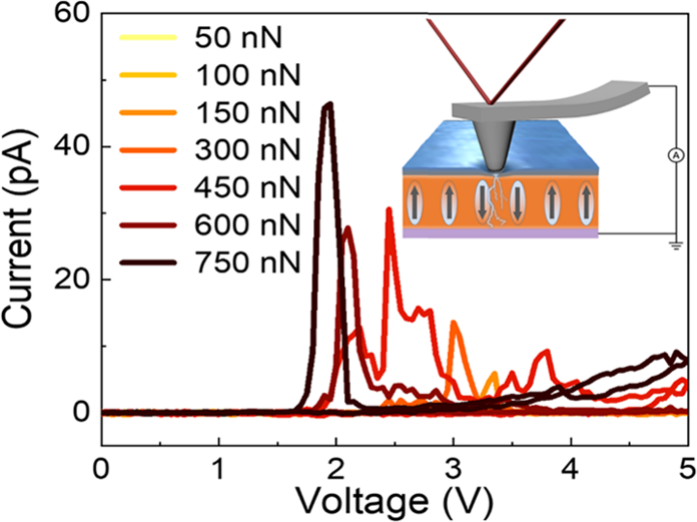

When scaling the lateral size of a ferroelectric random access memory (FeRAM) device down to the nanometer range, the polarization switching-induced displacement current becomes small and challenging to detect, which greatly limits the storage density of FeRAM. Here, we report the observation of significantly enhanced injection currents, much larger than typical switching currents, induced by polarization switching in BiFeO_3_ thin films via conductive atomic force microscopy. Interestingly, this injected current can be effectively modulated by applying mechanical force. As the loading force increases from ∼50 to ∼750 nN, the magnitude of the injected current increases and the critical voltage to trigger the current injection decreases. Notably, changing the loading force by an order of magnitude increases the peak current by 2–3 orders of magnitude. The mechanically boosted injected current could be useful for the development of high-density FeRAM devices. The mechanical modulation of the injected current may be attributed to the mechanical force-induced changes in the barrier height and interfacial layer width.

## 1 Introduction

Ferroelectric random access memory (FeRAM) and other ferroelectric-based nonvolatile memories in general use electrically switchable polarization states to store binary bits of information.^1−5^ FeRAM has attracted great interest because of its high endurance, fast read/write speed, and low power consumption.^6−9^ However, commercial FeRAM devices still possess relatively low memory densities due to scalability issues.^10^ One of the key factors limiting scalability is that the polarization switching-induced displacement current can be too small to detect when the cell area is very small (note: current is measured to characterize the polarization state in FeRAM).^11^ To enhance the displacement current, ferroelectric materials with large spontaneous polarizations (100–200 μC/cm^2^), such as BiFeO_3_ (BFO), Pb(Zr,Ti)O_3_, and PbTiO_3_,^12−14^ can be employed. However, even with these materials, the enhancement in displacement current remains limited. For example, Kwon et al.^15^ measured a displacement current on the order of only 10 pA in a BFO nanocapacitor with a lateral diameter of 300 nm. However, such small currents do not meet the requirement of a high signal-to-noise ratio for a high-density memory device.^16^ Therefore, exploring alternative mechanisms that could boost the current associated with polarization switching could have profound implications in terms of characterizing the polarization state and in turn overcoming the FeRAM scalability issue.

Recently, Li et al. reported the observation of an injected current induced by polarization switching in a Ga-doped BFO film with a 200 nm top electrode. Notably, the injected current was reported to be more than 2 orders of magnitude larger than the displacement current (typically a few pA), which provides a promising way to downscale FeRAM devices.^17^ Li et al. further revealed that the injected current may originate from a polarization switching-induced change in the barrier height of the nonferroelectric interfacial layer (IL) existing between the electrode and the ferroelectric layer. However, if the initial barrier height of the IL is very large, it can impede the charge transfer and thus make the injected current much smaller than expected.^18−20^ One intuitive solution is the careful engineering of the IL during the fabrication of the ferroelectric layer and the electrode,^21^ which, however, makes the fabrication process tedious and error prone. In exploring an alternative solution, Das et al. reported that the tunneling current across an ultrathin (11 unit cells) dielectric film can be systematically modified by applying mechanical force with an atomic force microscope (AFM) tip due to the flexoelectric control of the tunneling barrier profile.^22^ Since the IL of interest in a ferroelectric film is essentially a several-unit-cell-thick dielectric layer (see evidence in ref [^15^]) of similar thickness as that in the work of Das et al., we propose that application of mechanical force may be an effective approach to modulate the interfacial barrier and thus boost the injected current in BFO films.

To demonstrate this idea, we use a ∼50 nm BFO film as a model system and measure the current–voltage (*I*–*V*) characteristics under different loading forces applied by an AFM tip. It is observed that as the loading force increases, the magnitude of the injected current increases and the critical voltage to trigger the current injection decreases alongside the coercive fields for switching. Possible mechanisms are discussed in terms of the mechanical modulation of the barrier height and width of the IL.

## 2 Materials and Methods

Epitaxial BFO/Ca_0.96_Ce_0.04_MnO_3_ (CCMO) bilayers were fabricated on LaAlO_3_ (LAO) (001) substrates using pulsed laser deposition with a KrF excimer laser (λ = 248 nm). The target-to-substrate distance was set at ∼5.5 cm, and the laser fluence and repetition rate were fixed at ∼0.63 J/cm^2^ and 8 Hz, respectively. The CCMO electrode layer was first deposited at 680 °C and then the temperature was increased to 700 °C for the growth of the BFO film. The oxygen pressure was kept at 15 Pa during the growth of both CCMO and BFO films.

The crystal structures were examined by X-ray diffraction (XRD; PANalytical X′Pert PRO) and transmission electron microscopy (TEM; Titan ETEM G2, Thermo Fisher Scientific). AFM and piezoresponse force microscopy (PFM) images were recorded on a commercial AFM (Cypher, Asylum Research). The PFM images were acquired using dual AC resonance-tracking mode with an AC voltage of 1.0 V near a resonance frequency of ∼350 kHz.^23^ Band excitation piezoresponse spectroscopy (BEPS)^24^ and conductive atomic force microscopy (C-AFM) were performed using another commercial AFM (MFP-3D, Asylum Research). Unless otherwise specified, the loading force for AFM, PFM, and C-AFM was ∼100 nN, which was much lower than that required for the structural phase transition (∼400 nN). A National Instruments module controlled via a LabView interface was used for BEPS measurements. Force–voltage characteristics were measured by performing BEPS as a function of force (up to ∼925 nN) applied to the sample surface via the AFM tip. Forces ranging from ∼50 to ∼925 nN in increments of ∼125 nN were applied. The DC voltages (*V*_DC_) were varied over 64 steps as 0 V → +10 V → 0 V → −10 V → 0 V in increments of 0.625 V, while the AC voltage (*V*_AC_) was kept at 1.0 V and ∼350 kHz. The resulting data were fitted to a simple harmonic oscillator model using custom MATLAB scripts. For all AFM-based experiments, conductive Pt/Ir-coated probes (PPP-EFM, nanosensors) with a spring constant of 2.0 ± 0.2 N/m (as calibrated via the Sader method^25^) and a resonance frequency of ∼77 kHz were used. The deflection sensitivity (50.1 ± 0.1 nm/V) of the cantilever was determined by measuring 10 force–distance curves on a glass slide. The loading force used in this work has an estimated error of ∼10% based on the spring constant and deflection sensitivity uncertainties.^26^ The voltage was defined to be positive when the tip was positively biased.

## 3 Results and Discussion

**Figure 1:**
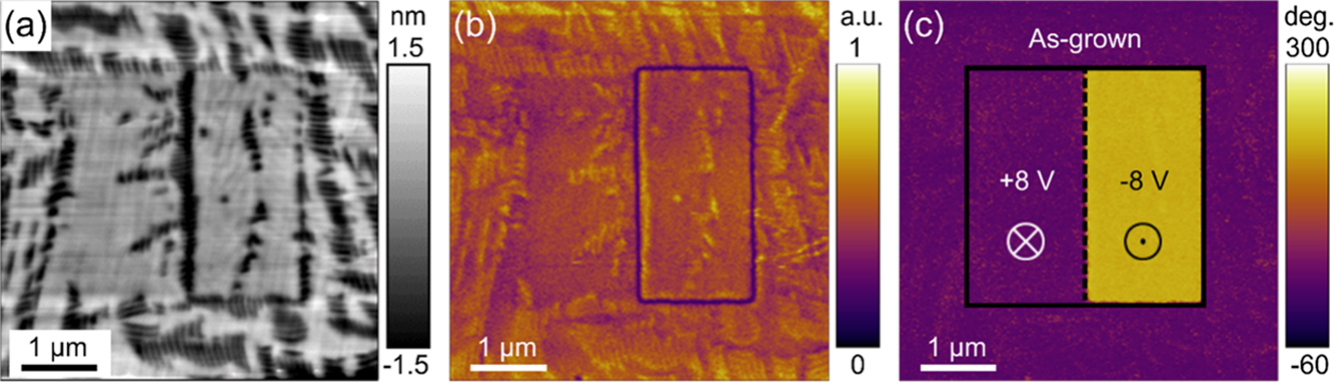
(a) Topography and vertical PFM (b) amplitude and (c) phase images after poling the mixed-phase BFO film grown on a CCMO-buffered LAO substrate with +8 V (left box) and −8 V (right box).

The topography of a typical ∼50 nm BFO film grown on the CCMO-buffered LAO substrate, showing distinct features of the BFO mixed phases, is presented in Figure 1a. Specifically, the flat regions are attributed to the tetragonal-like (T′) phase while the stripe-like regions correspond to the mixed T′ and rhombohedral-like (R′) phases. These topographic features are consistent with those of previously reported mixed-phase BFO films grown on the LAO substrates.^27^ When grown on the CCMO-buffered LAO substrate, the BFO film is subjected to a large compressive strain of 4.3% (note: the in-plane lattice constants of bulk BFO, CCMO, and LAO are ∼3.96, ∼3.77, and ∼3.79 Å, respectively).^28^ The large compressive strain can induce the formation of the T′ phase. As the film grows thicker (∼50 nm for our BFO film), the strain relaxes and hence the R′ phase, which is more stable than the T′ phase in the bulk form, is formed.^29,30^ The existence of mixed T′ and R′ phases can also be evidenced by the XRD and TEM results (see Figures S1 and S2), which display characteristic (00*l*) diffraction peaks from both T′ and R′ phases of BFO and display as bright (T′) and dark (R′) in Figure S2a. A mixed-phase BFO film was used in this work to show the universality of the mechanical modulation of current injection in different phases.

Vertical PFM amplitude and phase images were measured after poling the BFO film with +8 and −8 V applied to the left and right boxes (1.5 μm × 3 μm), respectively. As shown in Figure 1b,c, the ±8 V-poled regions show ∼180° phase contrast, and domain walls are observed at the boundaries of the −8 V-poled region, indicating that the domains in the ±8 V-poled regions are aligned in the opposite out-of-plane directions. In addition, it is deduced that the as-grown region has a downward polarization.

**Figure 2:**
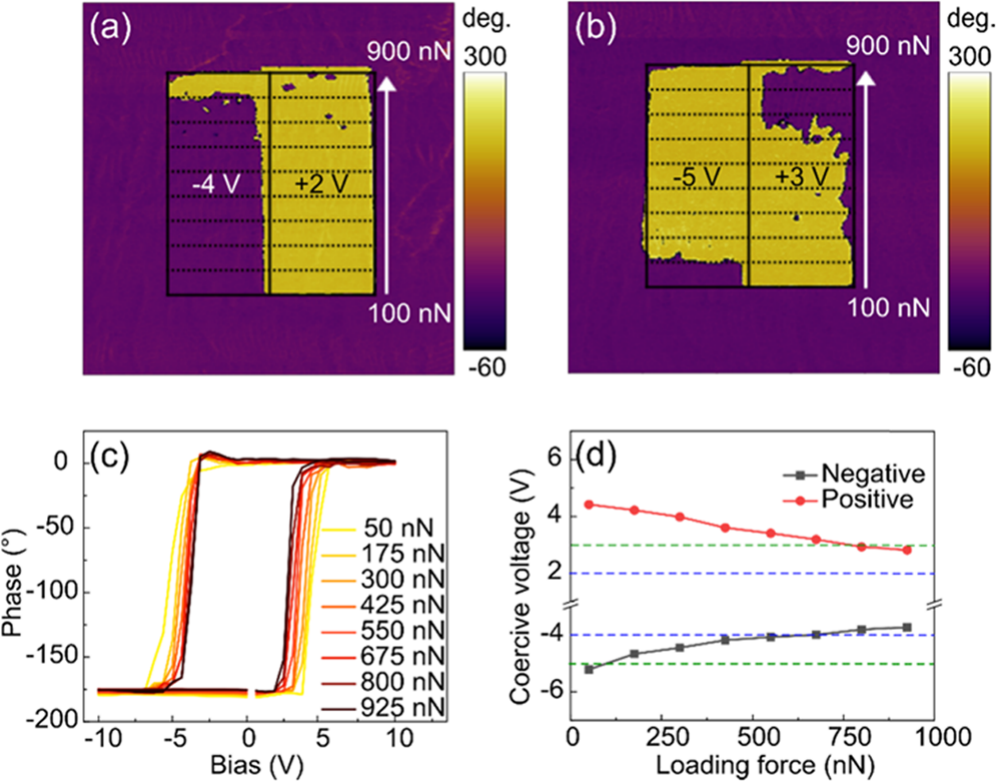
Vertical PFM (a, b) phase images after poling with tip biases of (a) −4/+2 V and (b) −5/+3 V and gradually increasing the loading force. The poled regions are the same as those in Figure 1b,c. (c) Average phase hysteresis loop under different loading forces obtained from BEPS measurements on 5 × 5 grids and (d) the coercive voltages (*V*_C_) obtained from (c). Blue and green dashed lines in (d) indicate the applied voltages in (a) and (b), respectively.

It has been reported that the mechanical force loading on a ferroelectric film can greatly influence domain switching.^31,32^ Therefore, the switching behavior of our BFO film needs to be further studied considering the effects of both the electric field and the mechanical force. First, the +8 and −8 V-poled regions (as shown in Figure 1c) were rescanned with tip biases of −4 and +2 V, which were less than the negative and positive coercive voltages under ∼100 nN, respectively, based on our preliminary measurements. As the tip was scanned from bottom to top, the loading force was increased from ∼100 to ∼900 nN in increments of ∼100 nN every ∼330 nm. The applied forces were determined by the product of the spring constant, deflection sensitivity, and tip deflection (see details in the Materials and Methods Section). Then, the resultant PFM phase images were measured, as shown in Figure 2a. At the tip bias of −4 V, no 180° domain (out-of-plane) switching occurs when the applied force is lower than ∼900 nN, but the domains can be fully switched when the applied force reaches ∼900 nN. By contrast, at the tip bias of +2 V, only partial domain switching occurs even when the applied force reaches ∼900 nN. Next, the tip biases were increased from −4/+2 V to −5/+3 V, respectively, and similar measurements were repeated. As shown in Figure 2b, the tip bias of −5 V (+3 V) can fully switch the domains at an applied force of ∼200 nN (∼700 nN). These results demonstrate that both positive and negative voltages required for downward and upward domain switching, respectively, decrease with increasing loading force.

To further investigate the effect of loading force on the domain switching, BEPS measurements with applied forces ranging from ∼50 to ∼925 nN were performed on 5 × 5 grids in the as-grown regions of the film. The resultant topography and PFM images after BEPS measurements, shown in Figure S3, demonstrate that no surface damage occurs even under ∼925 nN. Figure 2c shows the average hysteresis loops, which become narrower as the loading force increases. Despite the presence of a large compressive strain, which typically leads to an upward polarization, we observe an imprint favoring a downward polarization. The asymmetry of the ±*V*_C_ might then be associated with built-in fields associated with ferroelectric/electrode interfaces.^17,33^ The decrease in both positive and negative coercive voltages (determined in Figure S3) with increasing loading force is apparent in Figure 2d. One can also see from Figure 2d that at a critical voltage of −5 V (+3 V), the domain switching occurs when the loading force reaches ∼140 nN (∼650 nN), consistent with the results shown in Figure 2b. It is therefore confirmed that increasing mechanical force can decrease both positive and negative coercive voltages. At first glance, this result seems similar to previous reports on the mechanically induced flexoelectric effect in ferroelectric films.^31,32,34−36^ However, a different observation based on the phase-field modeling of the flexoelectric effect was reported previously, namely, the positive coercive voltage decreased while the negative coercive voltage increased with increasing mechanical force.^37^ The apparent discrepancy between our findings and previously reported observations suggests that mechanisms besides the flexoelectric effect may arise in our BFO film, as discussed later.

**Figure 3:**
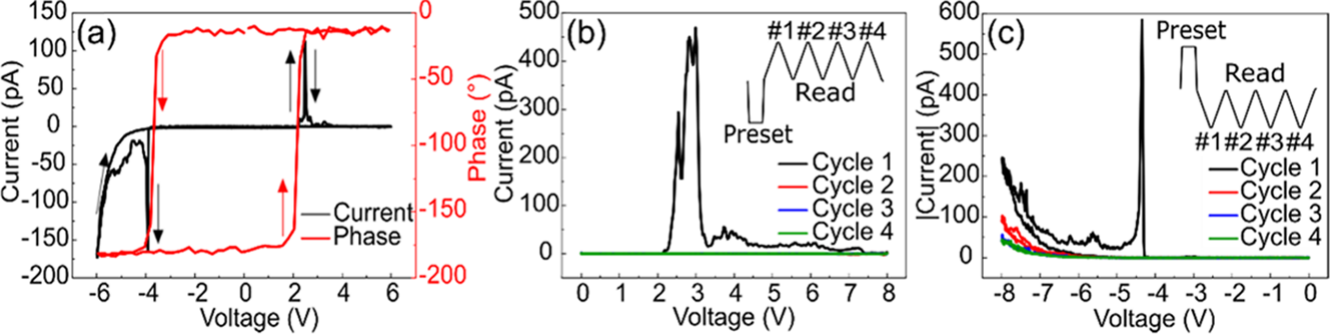
(a) Typical *I*–*V* curve measured under a loading force of 750 nN and the corresponding PFM phase loop. *I*–*V* curves measured with (b) 0 V → + 8 V → 0 V and (c) 0 V → −8 V → 0 V for four sequential cycles (absolute current is shown). Insets in (b, c) show the sequence of the applied voltage. Black and red arrows represent the voltage sweep direction for current and phase, respectively.

While the mechanical force influences the domain switching significantly, the question remains whether it can also modulate the conduction behavior. We therefore investigated the *I*–*V* characteristics by locating the tip on the as-grown film with different loading forces. Figure 3a presents the typical *I*–*V* curve under a loading force of ∼750 nN. One can clearly observe two current peaks located at −4 and +2.5 V. These voltages coincide with the coercive voltages, as shown in Figure 3a, suggesting that the current peaks are related to polarization switching.^12^ However, the current peak at −6 V represents the typical leakage behavior and is not directly relevant to polarization switching.^38^

To further distinguish whether a current peak is related to polarization switching or not, unipolar voltage sweeps were applied with the same 750 nN loading force. Figure 3b shows the *I*–*V* curves measured with 0 V → +8 V → 0 V for four sequential cycles. Prior to the first cycle, a preset pulse (−8 V, 4 s) was applied to switch the polarization to the upward direction. Only the black *I*–*V* curve, i.e., the first cycle, presents a large current peak at +3 V, while the other three subsequent cycles show no current peaks. For the unipolar negative voltage sweeps (see Figure 3c), the current peak at −4.5 V appears only in the first cycle, a behavior similar to that of the current peak at +3 V. However, there is another current peak at −8 V, which appears in every cycle. Because polarization switching occurs only in the first cycle and voltages of +3 and −4.5 V are close to the coercive voltages, the two current peaks at +3 and −4.5 V are thus confirmed to be polarization switching-related. It is also known that the leakage current exists in every cycle; therefore, the current peak at −8 V is attributed to the leakage current and is not directly relevant to polarization switching.

To further understand the nature of the current peak at the vicinity of the coercive voltage, the nominal polarization is calculated by integrating the current over time in the peak region (note: the leakage current was deducted using a positive up–negative down (PUND) method; see Figure S4). The calculated nominal polarizations are 979 and 212 mC/cm^2^ for the positive and negative switching, respectively, assuming a contact area with a radius of 25 nm. These nominal polarization values are 4 orders of magnitude larger than the polarizations of BFO (∼60 and ∼130 μC/cm^2^ for the R′ and T′ phases, respectively).^39,40^ Therefore, the polarization switching-induced displacement current can be excluded as the major origin for the current peak observed in our BFO film. Some previous works reported that band alignment can be modified by polarization, leading to nonvolatile resistive switching where the modified resistance state can persist even after the completion of polarization switching.^41^ However, here, we observe that the current is enhanced only during polarization switching while it drops significantly after the completion of polarization switching. Besides, the tip force-mediated migration of oxygen vacancies can also be excluded from the observed result because the low-resistance state turns into the high-resistance state quickly with increasing voltage at the same voltage polarity instead of maintaining the low-resistance state until the reverse migration of oxygen vacancies occurs at the opposite voltage polarity. The origin for the presence of the current peak during polarization switching may thus be attributed to the polarization switching-induced injected current as observed by Li et al.^17^ In brief, during polarization switching, the polarization charge can temporally induce a large electric field at the ferroelectric/electrode interface (several MV/cm),^42^ which reduces the barrier height and triggers a significant charge injection. Subsequently, some of the injected charge carriers may become trapped at the interface and screen the polarization charge. This can restore the barrier height and in turn results in the decrease of current.^20^ A current peak is therefore formed owing to the polarization switching-induced charge injection followed by charge trapping. This charge injection-mediated mechanism is supported by the observation of a higher current peak with increasing voltage sweep rate in Figure S5.

**Figure 4:**
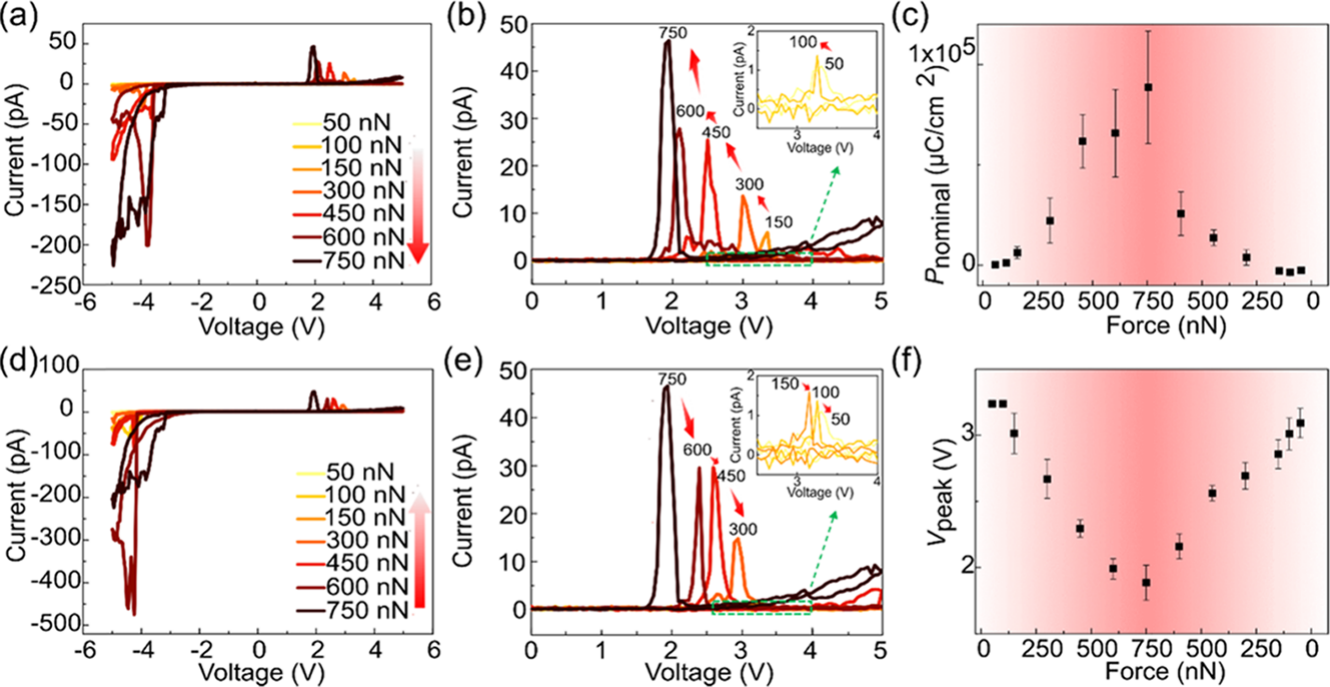
Typical *I–V* curves measured with (a) increasing and (d) decreasing loading forces on the BFO film. (b, e) *I–V* curves in the positive voltage region from (a) and (d), respectively. (c) Nominal polarization (*P*_nominal_) and (f) voltage corresponding to the current peak (*V*_peak_) as a function of loading force. The insets in (b, e) show the *I–V* curves at low forces in the green dashed area; the red arrows indicate the order in which the forces are applied. The legends in (b, e) are the same as those in (a, d).

We now focus on the modulation of observed injection currents via control of the mechanical force. We measured a series of *I–V* curves with the loading force increasing from ∼50 to ∼750 nN (Figure 4a). As the negative branches of the *I–V* curves are significantly affected by the leakage currents, only the positive branches will be further analyzed (see Figure 4b). The current peak is weak under a small loading force and increases with increasing loading force. After the loading force reached ∼750 nN, it was gradually reduced to ∼50 nN and the corresponding *I–V* curves were measured to test the reversibility of the mechanical modulation. As shown in Figure 4d,e, the current peak gradually reduces as the loading force decreases from ∼750 to ∼50 nN. The nominal polarizations under different loading forces are plotted in Figure 4c. The nominal polarization increases (decreases) with increasing (decreasing) loading force, and it reaches a maximum value of ∼89 mC/cm^2^ under a loading force of ∼750 nN. The difference (around an order of magnitude) between this nominal polarization value and that in Figure 3 may be due to film inhomogeneity or a decrease in the actual contact area. In addition, Figure 4b,e also shows that as the loading force increases (decreases), the current peak shifts systematically to the left (right) along the voltage axis, signifying the decrease (increase) of the voltage to trigger the current injection (i.e., *V*_peak_ in Figure 4f). This trend for *V*_peak_ is similar to that of the coercive voltage (see Figure 2d), further verifying the correlation between the current peak and polarization switching. It is noteworthy that the mechanical modulation of the current peak can be observed in both the T′-phase and mixed-phase regions, demonstrating the universality of this phenomenon (see Figure S6). The current peak increases with increasing force well below 400 nN (the threshold force of stress-mediated phase transitions) and the absence of the current peak in the *I*–*V* curves of unipolar cycles 2–4 at 750 nN (where such transitions could occur) in Figure 3b,c indicates that stress-mediated phase changes are not the driving mechanism for the enhancement of mechanically enhanced switching currents in our studies. Moreover, Figure S7 demonstrates that the enhancement of the current peak with increasing loading force is unlikely to be caused by the contact area change as the leakage current does not vary with the same trend as the current peak. In addition, the current peaks remain relatively stable after switching the device for 50 000 cycles using alternating 6 and −6 V pulses (pulse width: 0.5 ms) under a loading force of 750 nN, demonstrating good endurance of polarization switching and associated current injection (Figure S8).

**Figure 5:**
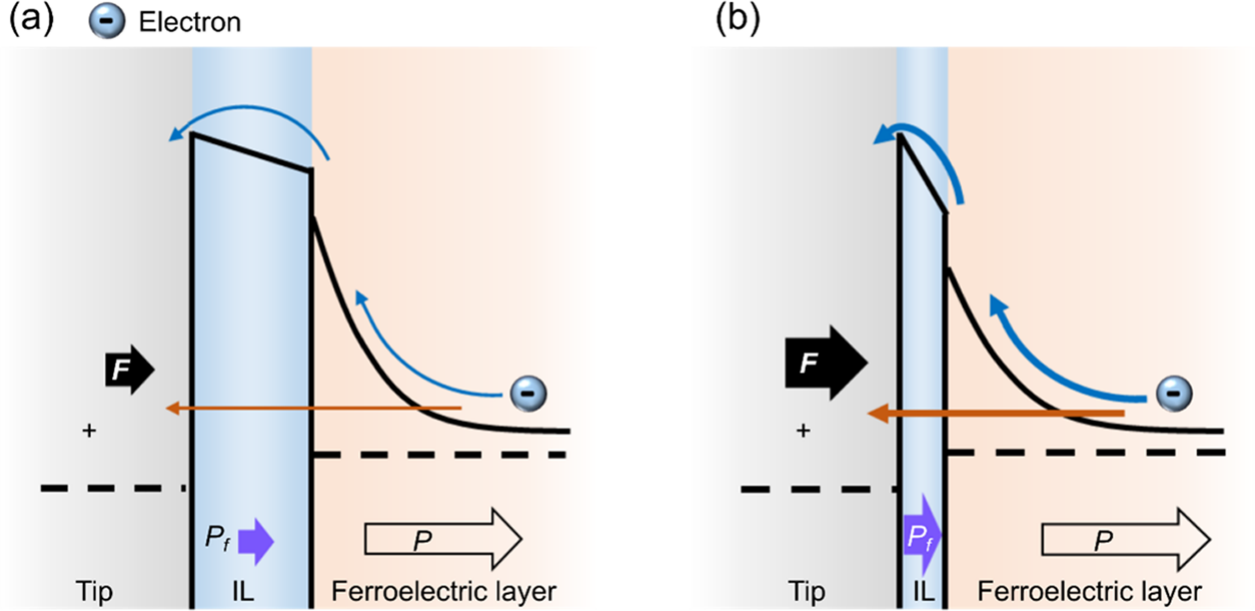
Schematic illustrations of the mechanism for the mechanical modulation of current injection in a ferroelectric film. Energy band diagrams of the metal-coated tip/interfacial layer (IL)/ferroelectric (FE) structure at a positive tip bias under (a) weak and (b) strong loading forces (solid black arrows). The orange and blue arrows indicate tunneling and thermionic emission, respectively. The purple arrows indicate flexoelectric polarization, while the arrows with black outlines indicate ferroelectric polarization.

The above results demonstrate that applying mechanical force can enhance the current peak as well as reduce the corresponding voltage (*V*_peak_). The mechanism may be illustrated by the schematic model shown in Figure 5. When the applied loading force is small, the effective barrier height of the IL between the tip and the BFO film is relatively high. Thus, the current flowing across the barrier, either by thermionic emission or tunneling, is low. When the loading force becomes large, the effective barrier height may be reduced due to the flexoelectric effect.^22^ The large loading force can also squeeze the interface layer (a rough estimation of the squeezed width is given in the Supporting Information), which is directly under the tip, as calculated by the finite element method, effectively narrowing the barrier width.^31,43^ Both the reduced barrier height and the narrowed barrier width can contribute to the enhanced current. In addition, the narrowed barrier width may reduce the voltage drop across the IL, and thus, the actual voltage across the ferroelectric layer may increase. This can be supported by the observation of the increase of amplitude response with loading force (Figure S3h).^44^ In addition, the mechanical force may flatten the energy barrier between two polarization states and thus results in a reduced coercive voltage. Therefore, a smaller applied voltage can trigger the polarization switching and associated current injection, thus reducing *V*_peak_.

## 4 Conclusions

The *I–V* characteristics of epitaxial BFO thin films on CCMO-buffered substrates were measured using C-AFM. Current peaks located at the vicinity of the coercive voltages were observed, and they were demonstrated to be the polarization switching-induced injected currents. We applied different mechanical forces via the AFM tip to modulate the current peak. By increasing the loading force, the current peak becomes higher and the voltage to trigger the current peak decreases, which may be explained by the reduced barrier height and the narrowed barrier width. We have therefore demonstrated an effective approach, i.e., applying mechanical force, to modulate the polarization switching-induced injected current. Since this injected current can be modulated to be many orders of magnitude larger than the displacement current, our finding may thus benefit the development of high-density FeRAM devices.

## Supporting Information Available

XRD θ–2θ scan of the BFO/CCMO/LAO epitaxial thin film (Figure S1); TEM images of the cross section of the BFO/CCMO/LAO substrate (Figure S2); BEPS measurements on the BFO film with different loading forces (Figure S3); positive up–negative down (PUND) waveform and *I–V* curve (Figure S4); *I*–*V* curves measured with different voltage sweep rates (Figure S5); *I*–*V* curves with increasing loading forces in a mixed-phase region and a T′-phase region (Figure S6); *I*–*V* curves with increasing loading force in one location (Figure S7); and *I–V* curves measured in the cycling test (Figure S8) (PDF)
