# Supplementary material for: Boosting Polarization Switching-Induced Current Injection by Mechanical Force in Ferroelectric Thin Films
Source: ACS Appl Mater Interfaces. 2021 May 26;13(22):26180–6. doi: 10.1021/acsami.1c04912 (PMC8289170; doi:10.1021/acsami.1c04912)
Supplement: Supplementary file 1 — am1c04912_si_001.pdf [file am1c04912_si_001.pdf]

Supporting Information

Boosting Polarization Switching-induced Current Injection by Mechanical Force in Ferroelectric Thin Films

Fengyuan Zhang[1,2,3], Hua Fan[4], Bing Han[5], Yudong Zhu[5], Xiong Deng[6], David Edwards[1,2], Amit Kumar[7], Deyang Chen[6], Xingsen Gao[6], Zhen Fan[6,*], and Brian J. Rodriguez[1,2,*]

[1]*School of Physics, University College Dublin, Belfield, Dublin D04 V1W8, Ireland*

[2]*Conway Institute of Biomolecular and Biomedical Research, University College Dublin, Belfield, Dublin D04 V1W8, Ireland*

[3]*Guangdong Provincial Key Laboratory of Functional Oxide Materials and Devices, Southern University of Science and Technology, Shenzhen 518055, People's Republic of China*

[4]*Institute for Quantum Science and Engineering, and Department of Physics, Southern University of Science and Technology, Shenzhen 518055, People's Republic of China*

[5]*Department of Materials Science and Engineering, Southern University of Science and Technology, Shenzhen 518055, People's Republic of China*

[6]*Institute for Advanced Materials and Guangdong Provincial Key Laboratory of Optical Information Materials and Technology, South China Academy of Advanced Optoelectronics, South China Normal University, Guangzhou 510006, People's Republic of China*

[7]*Centre for Nanostructured Media, School of Mathematics and Physics, Queen's University Belfast, UK*

Corresponding Author: Zhen Fan, E-mail: fanzhen@m.scnu.edu.cn

Brian J. Rodriguez, E-mail: brian.rodriguez@ucd.ie

I. Supplementary figures

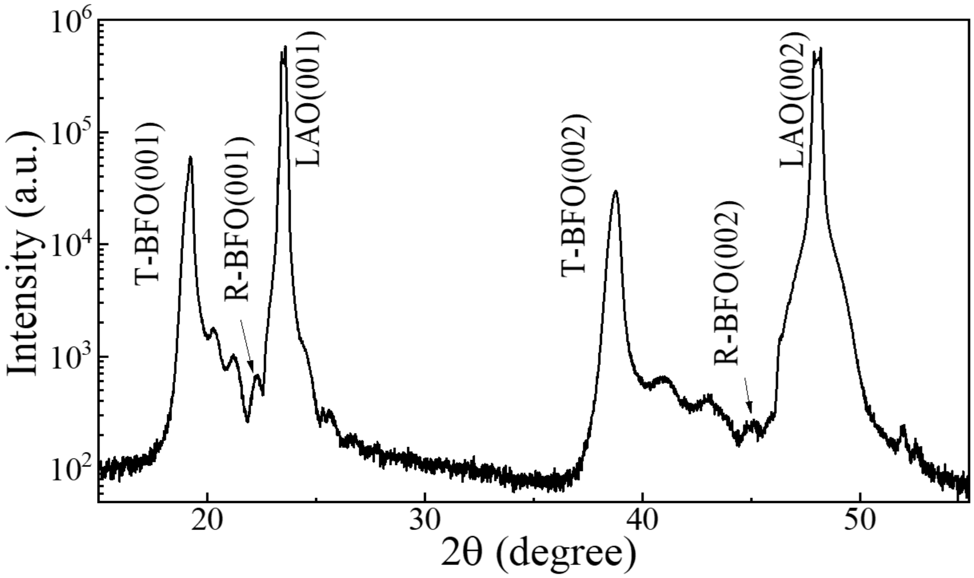

Figure S1. XRD *θ*–2*θ* scan of the BFO/CCMO/LAO epitaxial thin film. The peaks at ~19.2° and ~22.3° correspond to the T' and R' phases, respectively. The other two unmarked peaks at ~20.3° and ~21.2° may correspond to two transitional monoclinic phases that emerge along with the transition from the T' phase to the R' phase.

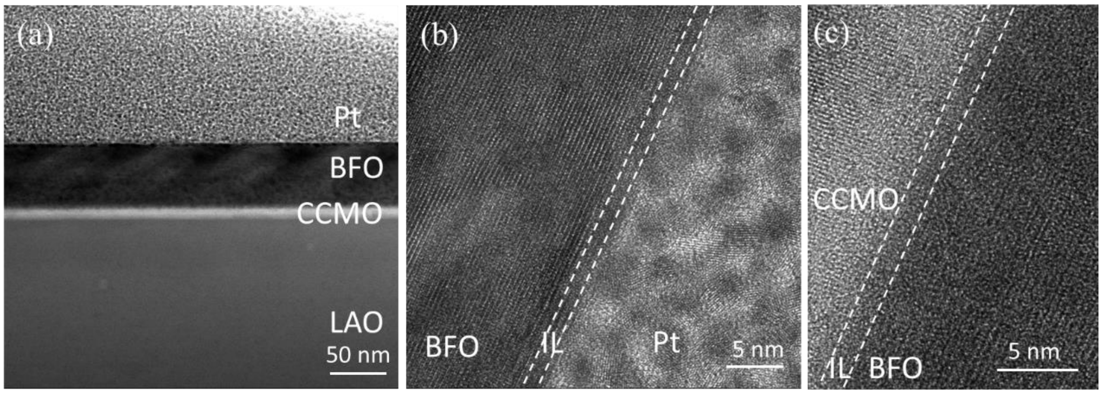

Figure S2. (a) Dark-field TEM image of the cross-sectional area of a mixed phase region in a BFO thin film on a CCMO-buffered (001) LAO substrate. High resolution bright-field TEM images of the cross-section of (b) the BFO top surface (top interface layer (IL) is in between Pt and BFO layers) and (c) the BFO bottom surface (bottom IL is in between BFO and CCMO layers). Both top and bottom ILs are ~1 nm and the CCMO layer is ~10 nm from the TEM images.

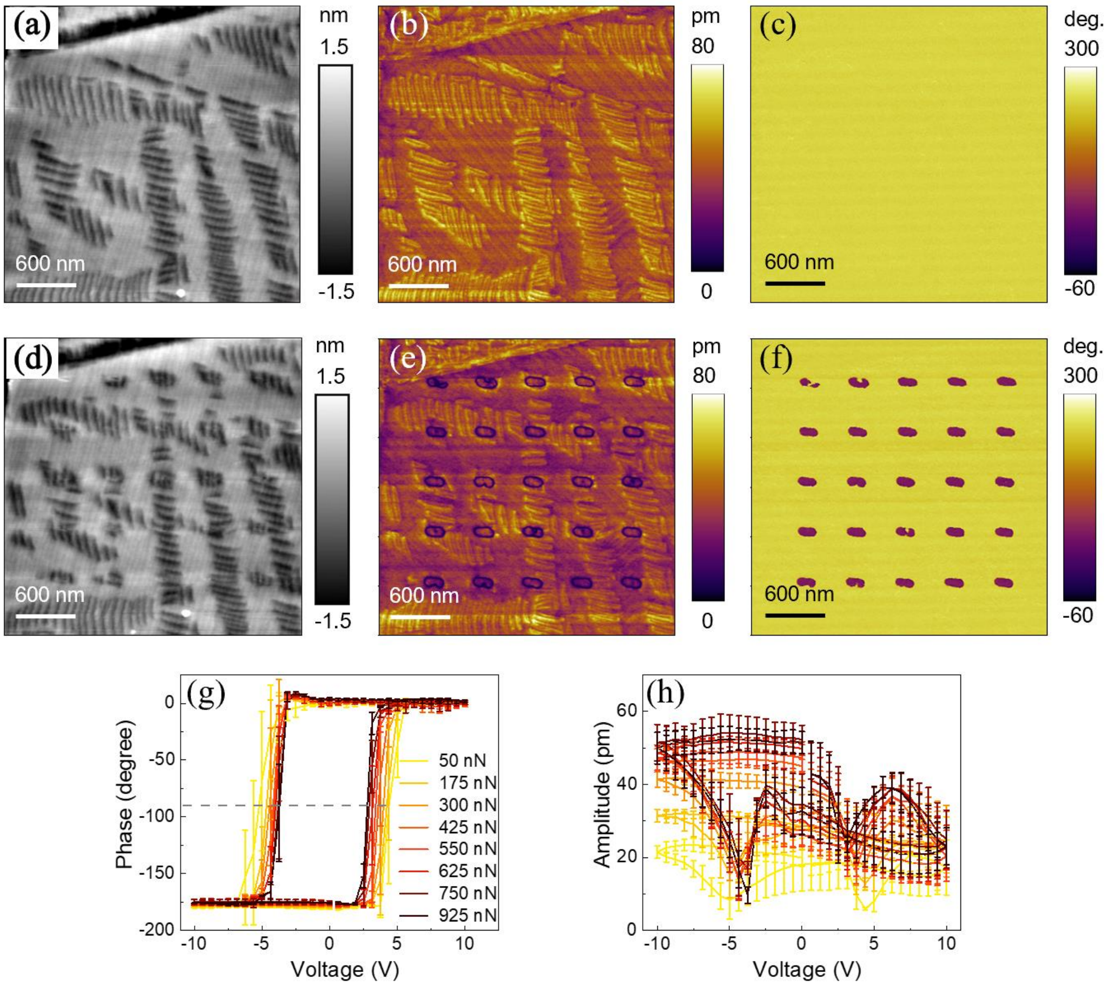

Figure S3. (a, d) Topography and PFM (b, c) amplitude and (c, f) phase images (a–c) before and (d–f) after BEPS measurements. Force was increased from 50 nN to 925 nN and back in 125 nN nm steps. DC voltages of 0 V → +10 V → 0 V → -10 V → 0 V in 0.625 V steps were applied at each applied force along with an AC voltage of 1 V. Comparing (a) and (d), no topography damage is observed after application of force. The PFM amplitude and phase images in (e) and (f), respectively, show domain switching after BEPS measurements. Averaged (g) phase and (h) amplitude hysteresis loops of 25 points (5 × 5 grids) at each applied force from 50 nN to 925 nN. The coercive voltages are determined from (g) at the intersects of the loops on the -90-degree axis (gray dashed line). The legends in (g) apply also to (h). The changes in the nose-like features arising in amplitude loops, that could be related to stress-induced phases transitions or ferroelastic wall motion, are relatively small enough to suggest that the contribution of such transitions to the overall hysteresis behavior (and particularly the coercive voltages) is relatively minor.

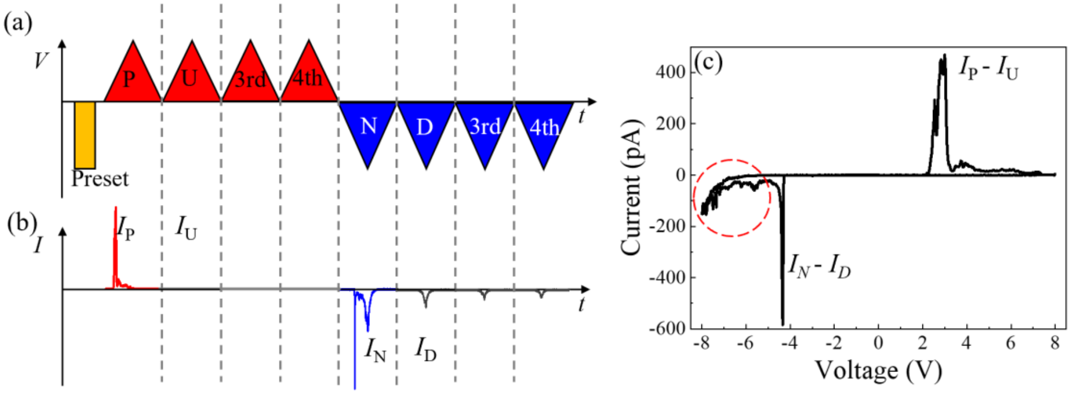

Figure S4. (a) Modified positive up–negative down (PUND) waveform. (b) The currents measured in the positive, up, negative, and down pulses are denoted as I_P, I_U, I_N, and I_D, respectively. (c) I–V curves of I_P–I_U and I_N–I_D, which were used to calculate the positive and negative nominal polarizations, respectively. Note that only the current in the peak region was integrated over time to calculate the nominal polarization, while the leakage current, which was not fully deducted by the PUND method (see red dashed circle), was manually excluded in the calculation. The whole cycle including 2000 points and the duration of each pulse is 2 ms. A contact area having a 25 nm radius is assumed (tip radius is ~25 nm); the nominal polarization is 2–3 orders of magnitude higher than the polarization of BFO even if the contact radius is 100 nm.

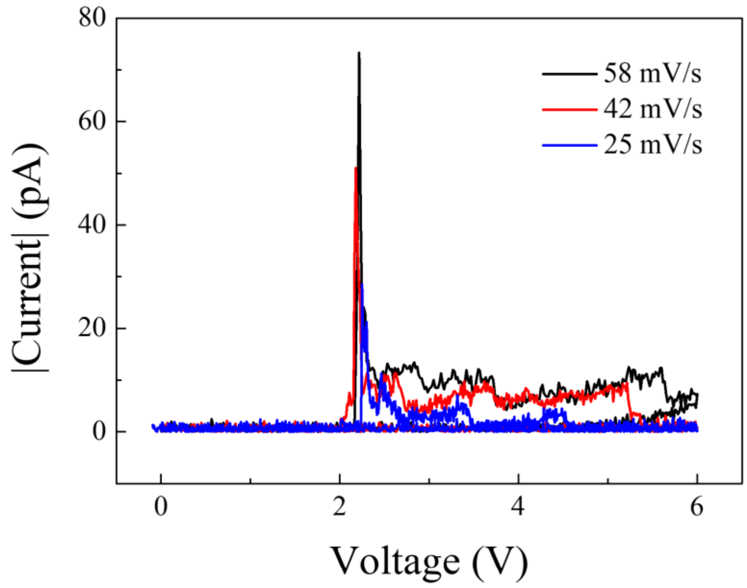

Figure S5. I–V curves measured with different voltage sweep rates at 750 nN.

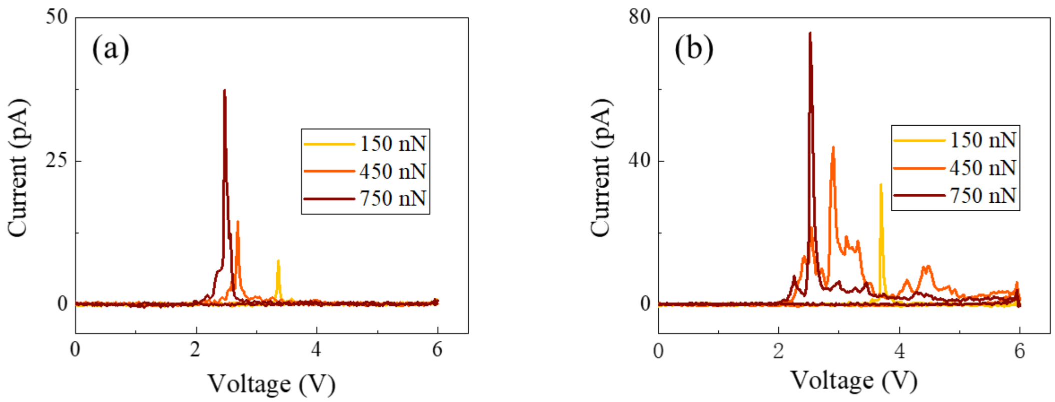

Figure S6. *I*–*V* curves with increasing loading forces in (a) a mixed-phase region and (b) a T'-phase region. In both regions, the current peak increases and shifts to lower voltage with increasing loading force. Though the phase can change from T' to R' above a certain force (~400 nN), a similar trend is observed in both locations.

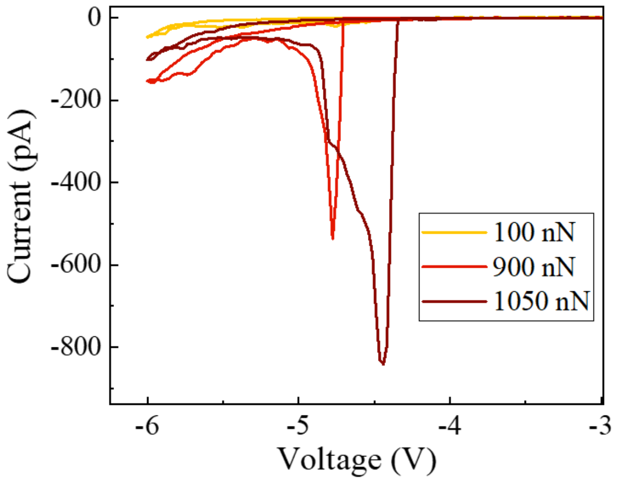

Figure S7. *I*–*V* curves with increasing loading force in one location. As the loading force increases from 900 nN to 1050 nN, the current peak becomes higher while the leakage current does not. Even though the leakage current at 900 nN is higher than at 100 nN, the current peak enhancement is much larger than that of the leakage current. Therefore, the enhancement of the current peak cannot be simply explained by the contact area change.

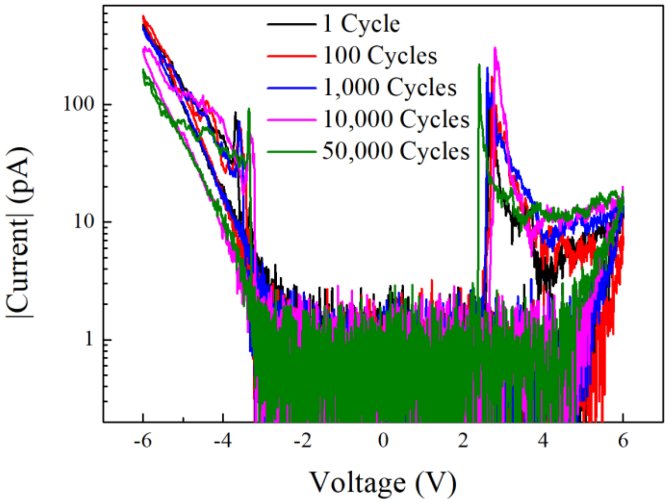

Figure S8. $I-V$ curves measured following cycling. Alternating 6 V and -6 V pulses (pulse width: 0.5 ms) were applied to switch the BFO film at 750 nN and the *I-V* curves were recorded after the specified number of cycles. The current peaks remain relatively stable after 50,000 cycles, demonstrating good endurance of polarization switching and associated current injection.

II. Estimation of the width change of the interface layer

A rough estimation is performed with the following equations and parameters:

$$E = \frac{\sigma}{\varepsilon} \quad (1)$$

$$\sigma = \frac{F}{A} \quad (2)$$

$$\varepsilon = \frac{\Delta L}{L} \quad (3)$$

with a loading force (F) of 1000 nN, a contact area (A) radius of 25 nm (i.e., the tip radius), and a Young's modulus (E) of 100 GPa.[1, 2] The estimated compressive strain (ε) is 0.005. With the BFO thickness (L) of ~50 nm extracted from the TEM image (Figure S2), the estimated width change (ΔL) of the IL is around 0.25 nm. However, because the tip typically has a spherical shape, the applied pressure could be higher. Thus, a width change of 0.25 nm is a conservative lower limit.

References

(1) Zhang, J.; Ke, X.; Gou, G.; Seidel, J.; Xiang, B.; Yu, P.; Liang, W. I.; Minor, A. M.; Chu, Y. H.; Van Tendeloo, G.; Ren, X.; Ramesh, R., A Nanoscale Shape Memory Oxide. *Nature Communications* **2013**, *4*, 2768.

(2) Li, Y. J.; Wang, J. J.; Ye, J. C.; Ke, X. X.; Gou, G. Y.; Wei, Y.; Xue, F.; Wang, J.; Wang, C. S.; Peng, R. C.; Deng, X. L.; Yang, Y.; Ren, X. B.; Chen, L. Q.; Nan, C. W.; Zhang, J. X., Mechanical Switching of Nanoscale Multiferroic Phase Boundaries. *Advanced Functional Materials* **2015**, *25*, 3405-3413.
